# Breast conserving surgery versus mastectomy: the effect of surgery on quality of life in breast cancer survivors in Malaysia

**DOI:** 10.1186/s12905-023-02738-w

**Published:** 2023-11-16

**Authors:** Maznah Dahlui, Meram Azzani, Nur Aishah Taib, See Mee Hoong, Suniza Jamaris, Tania Islam

**Affiliations:** 1 Centre of Population Health, Department of Social and Preventive Medicine, Faculty of Medicine, University of Malaya, Kuala Lumpur, 50603 Malaysia; 2 Faculty of Public Health, Universitas Surabaya, Surabaya, Indonesia; 3 Department of Public Health Medicine, Faculty of Medicine, Universiti Teknologi MARA, Sungai Buloh,Selangor, Malaysia; 4 Centre of Occupational Safety, Health and Wellbeing, Universiti Teknologi MARA, Puncak Alam, Selangor, Malaysia; 5 Department of Surgery, Faculty of Medicine, University of Malaya, Kuala Lumpur, 50603 Malaysia; 6 University Malaya Cancer Research Institute (UMCRI), Kuala Lumpur, Malaysia

**Keywords:** Breast cancer, Breast conserving surgery, Mastectomy, Quality of life, Survivors

## Abstract

**Background:**

In the competitive health care environment, patient satisfaction and quality of life (QoL) have become the subject of interest to evaluate the efficacy of therapeutic interventions as we experience improved breast cancer survival in modern times. The knowledge of the long-term effects of surgery on the QoL in breast cancer patients is currently limited in the Asian setting. The purpose of this longitudinal study is to evaluate the QoL of early-stage breast cancer patients undergoing mastectomy and breast-conserving surgery (BCS).

**Methods:**

In this prospective cohort study, the QoL of 208 patients who underwent mastectomy and the BCS treatment were assessed, using the European Organization for Research and Treatment of Cancer Core Quality of Life Questionnaire. The questionnaire was administered at the baseline, 6 and 12 months following diagnosis. One-way ANCOVA was used for statistical analysis.

**Results:**

A total of 208 female survivors of Stage 0–II breast cancer were included, among them 47.1% underwent BCS and 52.9% underwent mastectomy. Older (63.3%), Chinese women (63.6%), and patients with primary education (71.7%) were more likely to undergo mastectomy. At baseline, no significant differences were observed for QoL in both treatment groups. At 6 months, patients who underwent BCS had better social functioning scales( *P* = 0.006) and worse symptom scales for dyspnoea (*P* = 0.031), compared to mastectomy patients. One year after diagnosis, the role functioning score of the mastectomy group was significantly higher than the BCS group, specifically among patients who had undergone chemotherapy (*P* = 0.034).

**Conclusion:**

Patients who underwent BCS had better social functioning and worse dyspnoea symptoms compared to patients undergoing mastectomy at six months. During one year, there were only significant improvements in the role functioning among the mastectomy groups compared to the BCS groups. After further stratification, only mastectomy patients who received chemotherapy exhibited improved role functioning compared to patients those who did not undergo chemotherapy. Providing social and physical support postoperatively and monitoring patients for cancer worry, or other symptoms in the long-term survivorship period would be important to ensure optimal QoL.

## Background

Globally, breast cancer is the most common cancer among women and is the second leading cause of cancer-related deaths. Encouragingly, improvement to diagnostic and treatment facilities dedicated for breast cancer helped to reduce the mortality rates among patients [1]. In Malaysia, breast cancer is the most common cancer in women with 32.9% of newly diagnosed cancer cases in 2020 [2].

In the late 1890s, Halsted established the radical mastectomy as a standard treatment for breast cancer [3]. Whereas, in the early 1980s, large randomized studies were first proved that breast-conserving surgery (BCS) followed by postoperative radiotherapy was a valid alternative therapeutic to radical mastectomy in women with early breast cancer [4–6]. This modification was based on the result of prospective randomized studies, whereby survival rate is not correlated with either conserving surgery or mastectomy [4, 5, 7]. BCS is the usual choice for patients with early‐stage breast cancer. While some of the patients prefer mastectomy because of the fear of recurrence [6]. BCS remains a common choice for patients who prefer conserving treatment to maintain their body image [8–10].

In current times, within the rising competitive healthcare environment, patients’ satisfaction and quality of life (QoL) have become an area of interest to evaluate the efficacy of therapeutic interventions [5, 11]. Similarly, in cancer patients, the psycho-social factors and QoL have become important indicators used by the healthcare providers caring for these patients. The knowledge about QoL in breast cancer patients is derived from provisional studies whose results may differ according to country, culture, ethnicity, and societal relations. To the best of our knowledge, longitudinal studies on the QoL of Malaysian patients treated with BCS or mastectomy have not been reported in the literature. Therefore, this study aims to compare the impact of mastectomy versus the BCS on the QoL of breast cancer patients at different points of time during their survivorship period.

## Methodology

### Study design and methods

This study is a part of a prospective cohort study called the Malaysian Breast Cancer Cohort (MyBCC). MyBCC aims to determine the association between socio-demographic, lifestyle, and psychosocial factors, QoL as well as overall survival of multi-ethnic breast cancer survivors. The protocol of the MyBCC study can be found elsewhere [12].

The MyBCC study is an ongoing hospital-based prospective cohort study of Malaysian women who are newly diagnosed with primary breast cancer (within 3 months of diagnosis), above 18 years old, and able to read and understand Malay, English, Mandarin, or Tamil. The exclusion criteria were patients with a prior history of any other cancer, bedridden at the time of recruitment, and whose attending physician had certified them as unfit as a result of other prevailing medical conditions. Patients were recruited from February 2016 to December 2019.

The objective and details of the research were explained to all participants, and subjects who had provided their written informed consent of participation were included. Purposive sampling was used and a total of 208 MyBCC patients who underwent surgery and with complete information about their QoL, were included. Patients with advanced stages of cancer (3 and 4) and male patients were excluded.

### Procedure and measures

All questionnaires administered to the participants were done by trained research coordinators at the time of diagnosis (baseline). Socio-demographic data and clinical information were collected from the breast cancer registry database. The stage of the patient's breast cancer was confirmed by the surgeon using the American Joint Committee on Cancer (AJCC) staging method. The patient's surgical details were retrieved from the UMMC i-Pesakit (electronic database) records. In addition, the QoL was measured again at 6 and 12 months following diagnosis. Six months was chosen because the side effects of chemotherapy and radiation would have diminished, and after 1 year they had settled into long-term survivorship after completion of the cancer treatment.

The QoL was assessed using the European Organization for Research and Treatment of Cancer Core Quality of Life Questionnaire (EORTC QLQ-C30 and EORTC QLQ-BR23). Two modules were translated to the local languages and validated. The validity and reproducibility of the EORTC questionnaire have been proven to be acceptable [13, 14]. The EORTC QLQ-C30 comprised 30 items including five functional scales (physical, role, emotional, cognitive, and social), nine symptom scales (fatigue, nausea and vomiting, pain, dyspnoea, insomnia, appetite loss, constipation, diarrhoea, and financial difficulties) and Global health status scale [15]. The QLQ-BR23 contains of 23 items of functional scale and symptom scale. The four-functional scale evaluates body image, sexual functioning, sexual enjoyment, and future perspective, while the four-symptom scale evaluates systemic therapy side effects, breast symptoms, arm symptoms, and being upset by hair loss [16]. All the domains except upset by hair loss (number of patients at baseline: 11, 6 months: 45, and 1 year: 18) and sexual enjoyment (number of patients at baseline: 36, 6 months: 26, and 1 year: 24) were not evaluated. This is because a limited number of patients filled out the hair loss and sexual enjoyment questionnaires, primarily due to the fact that the majority of individuals were not experiencing hair loss and were not sexually active during those specific time periods. Information extracted from the questionnaires was scored accordingly. The raw score for each subscale was calculated and subsequently linearly transformed to a level between 0 and 100 (standardized raw score) according to the guidelines of the EORTC scoring manual. A higher score for functional scale scores represents a high/healthy level of functioning [17]. A high score for the global health status/QoL represents high QoL. However, a high score for a symptom scale represents a higher level of symptom, indicating poor QoL. The scoring approach for QLQ BR 23 is identical in principle to that of the functional and symptom scales of the QLQ-30.

### Statistical analysis

Data were checked for the normality using the Shapiro–Wilk test. Descriptive statistics analysis was performed for demographic characteristics and socioeconomic status of cancer patients. The mean and standard deviation for all items in the QLQ-C30 and QLQ BR23 were calculated for the BCS and mastectomy patients. Categorical variables are shown as frequency and percentage, while continuous variables are presented as (mean ± standard deviation). Chi-square test was applied to identify the association between the different socio-demographic factors and types of surgeries. A T-test was conducted to show the mean differences for QoL between both groups (mastectomy vs. BCS) at baseline, 6 months, and 1 year. One-way ANCOVA was used to find the adjusted QoL measures according to type of surgery at 6 months and 12 months following diagnosis. Post hoc tests were carried out to identify which groups differed. Subgroup analysis was carried out for chemotherapy treatment. A *P*-value of < 0.05 was considered as statistically significant.

## Results

Ninety-eight patients who had undergone BCS and one hundred-ten mastectomized patients were included in the final analysis. Background demographic and socio-economical details of the subjects are shown in Table 1. There were significant differences for many of the socio-demographic factors including age group, ethnicity, marital status and education level, and occupation label based on the two types of surgery (*P* < 0.05). As expected, there was a significant difference between cancer stage and radiotherapy with type of surgery, as BCS patient routinely receive radiotherapy (*P* < 0.005). Chinese (63.6%) and Indian (57.9%), women were more likely to have mastectomy whereas Malay women preferred BCS (70%). Comparing the patients who underwent BCS, those who underwent mastectomy tended to be older, unmarried/widowed, less educated, and were not working.

**Table 1:** Socio-demographic and clinical characteristics of study participants according to type of surgery (*N* = 208)

Characteristics	BCS (*N* = 98) N (%)	Mastectomy (*N* = 110) N (%)	*P* values
**Age**			
≤ 50	40(63.4)	23(36.6)	< 0.01*
51–64	46(53.8)	49(46.2)	
≥ 65	12(36.7)	38(63.3)	
**Ethnicity**			
*Malay*	42(70.0)	18(30.0)	< 0.01*
*Chinese*	40(36.4)	70(63.6)	
*Indian*	16(42.1)	22(57.9)	
**Marital Status**			
* Married*	79(53.7)	68(46.3)	< 0.01*
*Others*	19(31.1)	42(68.9)	
**Education Level**			
*Primary*	15(28.3)	38(71.7)	< 0.01*
*Secondary and above*	83(53.5)	72(46.5)	
**Income Level (RM)**			
≤ *5000*	73(44.8)	90(55.2)	0.2
> *5000*	25(55.6)	20(44.4)	
**Occupation Status**			
*Working*	50(59.5)	34(40.5)	< 0.01*
*Not working*	48(38.7)	76(61.3)	
**Stage**			
*0*	13(68.4)	6(31.6)	< 0.01*
*1*	49(53.8)	42(46.2)	
*2*	36(36.7)	62(63.3)	
**Chemotherapy (184)**			
*No*	44(50)	44(50)	0.48
*Yes*	43(44.8)	53(55.2)	
**Radiotherapy (183)**			
*No*	11(14.5)	65(85.5)	< 0.01*
*Yes*	76(71.0)	31(29.0)	
**Hormone therapy (110)**			
*No*	21(53.8)	18(46.2)	0.55
*Yes*	34(47.9)	37(52.1)	

Categorical data are expressed as percentage. *P* values were calculated using the Chi-square test for categorical variables

*Abbreviation*: *BCS =* Breast conserving surgery

^*^Significantly different at *P* < .05

Table 2 presents the average scores of each QoL domain at different time points by surgery type. From the QLQ-C30 questionnaire, the general health status scores in the mastectomy group were higher than in the BCS group at baseline (74.3 ± 16.6 vs. 73.1 ± 16.3), 6 months (76.2 ± 13. vs.70.4 ± 1 6.4) and 1 year (73.8 ± 15.4 vs. 71.5 ± 15.8). In the functional scale, some of the domains (physical functioning, role functioning, and cognitive functioning), the mastectomy group’s QoL was slightly better than those of the BCS group at 6 months and 1 year. Simultaneously the mean scores for symptoms, particularly nausea and vomiting, insomnia, appetite loss, constipation, and financial difficulty, in the BCS group were higher than those in the mastectomy group during 6 months and 1 year. The scores for fatigue, pain, dyspnoea (except 6 months), and diarrhoea, showed a higher rating on the symptom scale for the patient who had undergone mastectomy than those who had undergone BCS. The QLQ-BR23 functional scales showed the mean scores for body image were higher in the BCS group than the mastectomy group at the baseline but lower than the mastectomy group at 6 and 12 months after diagnosis. Among the symptom scale, the scores for sexual functioning were higher in the mastectomy group at baseline, during 6-month and 1 year time points. In contrast, mean scores for future perspective were comparatively higher among the BCS group in all 3-time points. The mean scores for breast symptoms were higher among the mastectomy group compared to the BCS group at baseline, 6 months, and 1 year after diagnosis.

**Table 2:** The quality of life score according to type of surgery at diagnosis, 6 months and 12 months following diagnosis (*N* = 208)

QoL domains	Surgery Type	At diagnosis (Mean ± SD)	At 6 months (Mean ± SD)	At 12 months (Mean ± SD)
**QLQ-C30 Questionnaire**				
**General Health Status**^**a**^	Mastectomy	74.3 ± 16.6	76.2 ± 13.6	73.8 ± 15.4
	BCS	73.1 ± 16.3	70.4 ± 1 6.4	71.5 ± 15.8
**Functional scales**^**a**^				
Physical functioning	Mastectomy	93.9 ± 13.1	91.3 ± 10.7	89.1 ± 30.6
	BCS	93.3 ± 12.1	86.8 ± 14.1	88.6 ± 15.0
Role functioning	Mastectomy	91.1 ± 16.0	93.7 ± 14.4	93.5** ± **12.5
	BCS	92.1 ± 14.4	90.9 ± 17.1	91.7 ± 16.7
Emotional functioning	Mastectomy	79.1 ± 19.4	86.5 ± 18.8	88.2 ± 13.4
	BCS	83.1 ± 20.3	88.3 ± 17.9	86.3 ± 18.0
Cognitive functioning	Mastectomy	88.7 ± 15.2	86.7 ± 17.0	87.9 ± 14.7
	BCS	87.0 ± 16.3	82.5 ± 18.1	83.3 ± 17.9
Social functioning	Mastectomy	94.7 ± 14.4	93.3 ± 13.4	95.7 ± 11.7
	BCS	92.4 ± 16.8	93.6 ± 15.4	94.6 ± 13.1
**Symptom scales**^**b**^				
Fatigue	Mastectomy	14.8 ± 17.6	15.7 ± 16.8	18.1 ± 19.7
	BCS	13.4 ± 16.7	17.7 ± 20.7	15.8 ± 18.7
Nausea and vomiting	Mastectomy	3.4 ± 8.9	3.0** ± **8.4	1.8 ± 6.7
	BCS	4.0 ± 13.0	4.3 ± 14.5	1.9 ± 6.7
Pain	Mastectomy	12.9 ± 17.1	15.9 ± 18.8	17.0 ± 16.7
	BCS	9.8 ± 16.7	16.5 ± 19.6	16.9 ± 19.1
Dyspnoea	Mastectomy	9.5 ± 20.8	6.8 ± 16.5	7.1 ± 18.6
	BCS	3.9 ± 12.5	9.3 ± 9.2	6.9 ± 16.9
Insomnia	Mastectomy	20.7 ± 28.5	13.2 ± 24.2	13.9 ± 24.3
	BCS	22.7 ± 30.9	25.4 ± 29.5	21.8 ± 28.3
Appetite loss	Mastectomy	6.4 ± 15.6	5.7 ± 14.3	3.4 ± 11.2
	BCS	4.8 ± 15.5	10.0 ± 20.4	6.0 ± 15.9
Diarrhoea	Mastectomy	1.3 ± 6.6	2.7 ± 12.3	1.3 ± 6.6
	BCS	1.5 ± 10.4	1.5 ± 6.9	1.2 ± 6.2
Constipation	Mastectomy	4.7 ± 15.1	6.4 ± 14.0	2.0 ± 8.0
	BCS	6.0 ± 18.7	8.1 ± 18.7	6.3 ± 15.9
Financial difficulties	Mastectomy	6.4 ± 17.6	9.5 ± 19.7	7.4 ± 20.0
	BCS	9.3 ± 21.2	13.0 ± 23.5	11.8 ± 24.1
**QLQ-BR23 Questionnaire**				
**Functional scales**^**a**^				
Body image	Mastectomy	93.2 ± 15.3	94.1 ± 14.7	94.7 ± 15.5
	BCS	96.9 ± 10.9	90.7 ± 19.0	94.2 ± 15.7
Sexual functioning	Mastectomy	22.7 ± 27.2	19.2 ± 25.4	25.9 ± 26.9
	BCS	11.8 ± 19.2	7.5 ± 16.6	6.2 ± 16.7
Future perspective	Mastectomy	67.0 ± 32.2	71.7 ± 28.8	73.4 ± 27.8
	BCS	78.9 ± 25.4	76.1 ± 27.1	80.6 ± 23.6
**Symptom scales**^**b**^				
Systemic therapy side effects	Mastectomy	8.8 ± 11.6	11.5 ± 12.5	8.9 ± 9.9
	BCS	7.2 ± 11.8	14.4 ± 15.2	9.3 ± 10.1
Breast symptoms	Mastectomy	9.9 ± 17.1	11.3 ± 16.2	11.1 ± 13.3
	BCS	9.2 ± 18.6	9.9 ± 13.8	8.2 ± 10.3
Arm symptoms	Mastectomy	9.9 ± 17.1	10.1 ± 14.5	12.0 ± 17.4
	BCS	9.2 ± 18.6	12.7 ± 19.5	11.2 ± 17.1

Mean value between BCS and Mastectomy was evaluated by t test

*Abbreviations*: *SD* = Standard deviation, *BCS =* Breast conserving surgery

^a^Higher score indicates better functioning

^b^Higher score indicates more symptoms

Table 3 shows the adjusted (age, ethnicity, hormonal therapy, chemotherapy, radiotherapy and the quality of life score at time of diagnosis (baseline)) mean difference of QoL according to the types of surgeries at baseline, 6 and 12 months following diagnosis. The QLQ-C30 questionnaire at baseline showed no significant difference in general health status at different time points. There was no significant difference in QoL between the BCS and mastectomy patients in any of the four functional domains (physical functioning, role, functioning, emotional functioning and cognitive functioning). At 6 months after diagnosis, the BCS group's social functioning score (mean score 97.6 vs 88.6; F = 7.85 and *P* = 0.006) were significantly higher than mastectomy patients. However, the dyspnoea symptom scale of the mastectomy group was significantly lower (mean score of 2.5 vs. 12.5, F = 4.76, and *P* = 0.031) than that of the BCS, indicating better QoL. At 1 year after diagnosis, the role functioning score of the mastectomy group was significantly higher than the BCS group (mean score 94.8 vs. 85.7; F = 4.89, *P* = 0.029). From the QLQ-BR23 feedback, among the functional and symptom scales only sexual functioning was significantly higher among the mastectomy group compared to the BCS group at baseline (mean score 22.2 vs. 10.9; F = 5.2 and *P* = 0.027) and during six months (mean score 18.5 vs. 8.7; F = 25.1 and *P* = 0.000). Nevertheless during 1 year after diagnosis, there was no significant difference.

**Table 3:** Adjusted quality of life measures of EORTC QLQ-C30 and EORTC QLQ-BR23 scales according to type of surgery at 6 and 12 months following diagnosis (*N* = 208)

Quality of Life Domain	Type of surgery	At baseline			At 6 months^c^			At 12 months^c^		
		**Mean (CI)**	***F***	***P* value**	**Mean (CI)**	***F***	***P* value**	**Mean (CI)**	***F***	***P* value**
**QLQ-C30 Questionnaire**										
**General Health Status**^**a**^	Mastectomy	75.2(69.8–80.7)	1.512	0.222	75.0(70.4–79.6)	1.92	0.168	73.7(68.7–78.7)	0.825	0.366
	BCS	69.8(64.2–75.3)			69.9(65.3–74.5)			70.0(65.0–75.0)		
**Functional scales**^**a**^										
Physical functioning	Mastectomy	91.3(86.8–95.8)	0.175	0.677	89.6(85.2–94.1)	2.08	0.152	91.1(87.0–96.8)	3.367	0.069
	BCS	92.8(88.3–97.3)			84.4(79.9–88.9)			84.6(79.7–89.5)		
Role functioning	Mastectomy	88.7(83.5–93.9)	0.777	0.380	91.3(85.9–96.6)	0.01	0.906	94.8(89.7–100.0)	4.894	0.029*
	BCS	92.4(87.2–97.6)			90.8(85.4–96.1)			85.7(80.5–90.8)		
Emotional functioning	Mastectomy	82.4(76.4–88.4)	0.854	0.357	85.2(79.3–91.1)	0.38	0.537	89.1(83.6–94.6)	1.902	0.171
	BCS	77.9(72.0–83.9)			88.2(82.3–94.1)			82.9(77.4–88.4)		
Cognitive functioning	Mastectomy	88.3(82.7–93.9)	0.211	0.647	84.1(78.2–90.0)	0.02	0.868	85.3(79.6–90.9)	0.640	0.425
	BCS	86.2(80.6–91.8)			83.3(77.5–89.2)			81.6(75.9–87.3)		
Social functioning	Mastectomy	96.0(90.4–101.6)	2.797	0.097	88.6(84.7–92.6)	7.85	0.006*	95.7(91.7–99.6)	0.067	0.796
	BCS	88.4(82.8–94.0)			97.6(93.7–101.6)			94.8(90.9–98.8)		
**Symptom scales**^**b**^										
Fatigue	Mastectomy	16.2(10.1–22.2)	0.040	0.843	13.5(7.5–19.4)	3.39	0.068	18.6(12.1–25.0)	0.002	0.960
	BCS	15.2(9.0–21.4)			22.4(16.4–28.3)			18.8(12.3–25.4)		
Nausea and vomiting	Mastectomy	4.6(0.3–8.9)	0.018	0.894	2.8(-1.8–7.6)	1.56	0.214	1.5(-1.2–4.2)	0.680	0.411
	BCS	5.0(0.7–9.3)			7.7(2.9–12.5)			3.3(0.6–6.0)		
Pain	Mastectomy	13.4(7.5–19.4)	0.571	0.451	16.0(9.6–22.4)	0.35	0.555	17.1(11.3–22.9)	0.314	0.577
	BCS	9.8(3.9–15.7)			19.1(12.7–25.4)			19.8(13.9–25.6)		
Dyspnoea	Mastectomy	11.6(5.0–18.2)	0.944	0.333	2.5(-3.0–8.2)	4.76	0.031*	7.6(1.4–13.8)	0.119	0.731
	BCS	6.5(-0.079–13.0)			12.5(6.9–18.2)			9.3(3.1–15.5)		
Insomnia	Mastectomy	17.2(7.5–26.8)	1.570	0.213	12.7(4.4–21.1)	2.55	0.113	13.5(4.7–22.4)	2.118	0.149
	BCS	27.0(17.3–36.6)			23.5(15.2–31.9)			23.9(15.1–32.8)		
Appetite loss	Mastectomy	8.1(2.5–13.7)	0.236	0.609	6.5(0.8–12.2)	1.17	0.281	3.7(-1.1–8.6)	0.741	0.391
	BCS	5.8(0.171–11.4)			11.5(5.8–17.3)			7.1(2.2–12.0)		
Diarrhoea	Mastectomy	2.5(-1.2–6.3)	0.011	0.919	3.8(0.0–7.6)	0.50	0.481	1.7(-0.6–4.1)	0.002	0.963
	BCS	2.8(-0.9–6.7)			1.6(-2.1–5.4)			1.8(-0.5–4.2)		
Constipation	Mastectomy	3.8(-2.9–10.6)	2.446	0.121	6.7(1.6–11.7)	0.53	0.468	3.6(-0.6–7.9)	1.077	0.302
	BCS	12.4(5.6–19.2)			9.6(4.6–14.6)			7.2(2.9–11.5)		
Financial difficulties	Mastectomy	2.7(-3.3–8.8)	3.762	0.055	10.3(3.4–17.2)	0.09	0.758	10.1(1.9–18.2)	0.361	0.549
	BCS	12.3(6.2–18.5)			12.0(5.1–18.9)			14.1(5.9–22.2)		
**QLQ-BR23 Questionnaire**										
**Functional scales**^**a**^										
Body image	Mastectomy	10.1(1.9–18.2)	0.361	0.549	92.7(87.9–97.6)	0.03	0.852	92.6(88.0–97.2)	1.240	0.268
	BCS	14.1(5.9–22.2)			92.0(87.1–96.9)			96.7(92.1–101.3)		
Sexual functioning	Mastectomy	22.2(15.1–29.4)	5.2	0.027*	18.5(15.7–21.3)	25.1	0.000*	14.4(8.9–19.9)	2.1	0.142
	BCS	10.9(3.8–17.9)			8.7(5.9–11.6)			7.9(2.4–13.4)		
Future perspective	Mastectomy	67.8(58.8–76.8)	1.01	0.315	69.6(62.1–77.2)	3.28	0.084	69.8(61.9–77.7)	2.7	0.098
	BCS	74.2(65.3–83.2)			79.1(71.3–86.9)			80.4(72.5–88.3)		
**Symptom scales**^**b**^										
Systemic therapy side effects	Mastectomy	8.9(5.5–12.3)	0.02	0.875	14.2(11.4–17.0)	2.4	0.139	10.6(7.2–13.9)	0.211	0.647
	BCS	9.3(5.9–12.7)			17.1(14.3–19.9)			11.8(8.4–15.2)		
Breast symptoms	Mastectomy	8.8(2.6–14.9)	0.31	0.531	13.1(7.8–18.3)	0.4	0.503	12.6(8.5–16.7)	1.1	0.297
	BCS	11.5(5.3–17.6)			10.2(4.9–15.5)			9.1(5.1–13.3)		
Arm symptoms	Mastectomy	8.7(2.5–14.9)	0.3	0.531	11.8(6.4–17.3)	0.1	0.731	12.3(6.4–18.1)	0.1	0.760
	BCS	11.5(5.3–17.6)			13.3(7.9–18.8)			13.7(7.9–19.5)		

*P* values were calculated by Analysis of covariance (ANCOVA)

*Abbreviation*: *BCS =* Breast conserving surgery

^*^significant at *P* < 0.05

^a^Higher score indicates better functioning

^b^Higher score indicates more symptom

^c^ANCOVA adjusted for age, ethnicity, hormonal therapy, chemotherapy, radiotherapy and the quality of life score at time of diagnosis (baseline)

Table 4 shows the subgroup analysis outcome based on chemotherapy treatment status (yes, no) of the adjusted mean difference of QoL, according to surgery type at baseline, 6 and 12 months following diagnosis. From the QLQ-C30 questionnaire, at baseline and 6 months after diagnosis there was no significant difference in the QoL functional and symptom scales between the BCS and mastectomy patients except dyspnoea. At 6 months, BCS groups (undergoing chemotherapy) were more dysphonic than mastectomy patients (mean score 15.0 vs. 0.0; F = 6.661, *P* = 0.012). However, one year after diagnosis, dyspnoea was insignificant also among the two groups and only the role functioning score of the mastectomy group was significantly higher than the BCS group, among patients undergoing chemotherapy treatment (mean score 95.2 vs. 84.4; F = 4.69, *P* = 0.034). For the QLQ-BR23 scoring, at baseline and 6 months after diagnosis among the functional and symptom scales, there was no significant difference except the mean scores of systematic therapy side effects. Patients undergoing chemotherapy BCS groups had more systematic therapy side effects compared to mastectomy patients (mean score 24.1 vs. 11.4; F = 6.5, *P* = 0.013). Nevertheless, there was no significant difference in 1 year except mastectomy patients who did not undergo chemotherapy had a higher sexual functioning mean score in contrast to the BCS group (mean score 25.8 vs. 2.4; F = 5.9, *P* = 0.02).

**Table 4:** Subgroup analysis of adjusted quality of life measures based on chemotherapy treatment (yes, no) according to type of surgery at 6 and 12 months following diagnosis (*N*= 208)

**Quality of Life Domain (based on chemotherapy treatment (Yes/No)**	**Type of surgery**	**At baseline**			**At 6 months**^**c**^			**At 12 months**^**c**^		
		**Mean (CI)**	***F***	***P value***	**Mean (CI)**	***F***	***P value***	**Mean (CI)**	***F***	***P value***
**QLQ-C30 Questionnaire**										
**General Health Status(no)**^**a**^	Mastectomy	71.8(60.7-82.8)	0.826	0.369	80.7(72.6-88.7)	3.245	0.079	74.3(64.7-83.8)	0.711	0.404
	BCS	63.0(51.2-74.8)			68.2(59.9-76.5)			67.3(57.5-77.2)		
**General Health Status(yes)**^**a**^	Mastectomy	79.4(73.2-85.5)	2.123	0.150	74.0(68.5-79.5)	1.933	0.170	74.1(67.8-80.4)	0.429	0.515
	BCS	72.4(66.4-78.5)			68.1(62.6-73.5)			71.0(64.8-77.1)		
**Functional scales**^**a**^										
Physical functioning (no)	Mastectomy	89.6(78.5-100.7)	0.040	0.842	88.0(81.1-94.9)	0.001	0.982	89.9(80.9-99.0)	0.309	0.581
	BCS	87.7(76.2-99.2)			87.9(80.7-95.0)			85.6(76.2-94.9)		
Physical functioning (yes)	Mastectomy	92.7(88.9-96.5)	1.259	0.266	90.5(84.4-96.9)	2.942	0.092	93.3(87.0-99.6)	3.602	0.063
	BCS	96.0(92.3-99.8)			82.3(76.2-88.4)			84.0(77.8-90.2)		
Role functioning (no)	Mastectomy	86.7(75.7-97.6)	0.097	0.757	85.5(75.2-95.9)	1.399	0.244	91.8(82.2-101.3)	0.032	0.859
	BCS	89.6(78.3-100.9)			96.1(85.4-106.7)			90.3(80.4-100.2)		
Role functioning (yes)	Mastectomy	90.4(84.7-96.2)	0.650	0.423	93.8(87.3-100.3)	0.995	0.323	95.2(88.7-101.7)	4.691	0.034*
	BCS	94.0(88.4-99.7)			88.8(82.4-95.2)			84.4 (78.0-90.7)		
Emotional functioning (no)	Mastectomy	81.7(69.8-93.6)	0.176	0.677	85.3(75.3-95.2)	1.151	0.290	89.3(79.9-98.8)	0.403	0.529
	BCS	77.4(65.0-89.7)			94.5(84.2-104.7)			84.2(74.4-93.9)		
Emotional functioning (yes)	Mastectomy	82.4(75.5-89.4)	0.454	0.503	84.0(76.0-91.9)	0.035	0.852	89.3(82.0-96.6)	1.812	0.183
	BCS	78.8(72.0-85.6)			85.1(77.3-92.9)			81.7(74.6-88.9)		
Cognitive functioning (no)	Mastectomy	84.9(73.6-96.1)	0.010	0.919	88.7(78.1-99.3)	0.924	0.342	89.8(81.4-98.2)	1.005	0.322
	BCS	83.9(72.3-95.5)			79.9(69.0-90.8)			82.5(73.8-91.2)		
Cognitive functioning (yes)	Mastectomy	90.9(84.1-97.6)	0.402	0.529	83.0(75.5-90.6)	0.006	0.936	82.9(74.9-91.0)	0.212	0.647
	BCS	87.6(80.9-94.2)			83.5(76.1-91.0)			80.1(72.2-88.0)		
Social functioning (no)	Mastectomy	96.5(83.2-109.8)	2.065	0.158	90.2(84.2-96.3)	3.134	0.084	95.9(89.9-101.9)	0.019	0.891
	BCS	80.0(66.3-93.8)			99.5(93.2-105.7)			96.6(90.4-102.8)		
Social functioning (yes)	Mastectomy	96.9(91.7-102.0)	1.036	0.313	88.0(82.5-93.5)	3.500	0.066	95.1(89.6-100.6)	0.066	0.798
	BCS	92.8(87.8-97.9)			95.9(90.5-101.3			94.0(88.6-99.4)		
**Symptom scales**^**b**^										
Fatigue (no)	Mastectomy	9.7(-1.4-21.0)	2.040	0.161	15.0(4.9-25.1)	0.003	0.958	19.9(8.4-31.3)	0.268	0.608
	BCS	23.6(12.0-35.2)			14.5(4.1-24.9)			14.7(2.4-26.9)		
Fatigue(yes)	Mastectomy	18.9(11.4-26.5)	1.696	0.198	14.1(6.1-22.1)	3.729	0.058	18.1(9.7-26.5)	0.215	0.645
	BCS	11.2(3.5-18.9)			26.0(18.2-33.8)			21.1(12.8-29.3)		
Nausea and vomiting (no)	Mastectomy	3.6(-6.1-13.4)	0.213	0.647	0.2(-10.6-11.1)	2.164	0.149	0.6(-6.0-7.3)	1.168	0.286
	BCS	7.5(-2.5-17.6)			14.1(2.8-25.4)			6.9(-0.0-13.8)		
Nausea and vomiting (yes)	Mastectomy	5.5(1.4-9.6)	0.575	0.451	3.7(-0.9-8.4)	0.047	0.830	1.1(-1.0-3.3)	0.175	0.677
	BCS	3.1(-0.8-7.1)			4.4(-0.1-9.1)			1.8(-0.2-4.0)		
Pain (no)	Mastectomy	17.3(4.7-30.0)	0.582	0.450	19.8(8.3-31.2)	0.646	0.426	15.6(4.6-26.6)	0.147	0.703
	BCS	9.8(-3.9-22.1)			11.8(0.0-23.7)			19.2(7.9-30.6)		
Pain (yes)	Mastectomy	11.9(5.2-18.6)	0.299	0.586	15.6(7.4-23.8)	0.897	0.347	17.2(10.0-24.4)	0.489	0.487
	BCS	9.1(2.5-15.6)			21.6(13.6-29.7)			21.1(14.0-28.2)		
Dyspnoea (no)	Mastectomy	14.2(3.1-25.2)	0.674	0.416	9.3(0.0-18.6)	0.238	0.628	9.4(-3.7-22.7)	0.001	0.978
	BCS	6.3(-5.0-17.7)			12.5(-4.1-15.0)			9.7(-3.8-23.4)		
Dyspnoea (yes)	Mastectomy	9.9(1.2-18.6)	0.250	0.619	0.0 (-7.4-7.6)	6.661	0.012*	5.4(-1.5-12.4)	0.674	0.415
	BCS	6.5(-1.9-15.0)			15.0(7.6-22.4)			9.8(3.0-16.6)		
Insomnia(no)	Mastectomy	11.3(-5.4-28.2)	2.983	0.092	9.9(-3.9-23.8)	1.185	0.283	11.4(-5.2-28.2)	0.464	0.500
	BCS	26.5(19.1-54.0)			22.9(8.6-37.3)			21.3(4.0-38.6)		
Insomnia(yes)	Mastectomy	19.7(7.5-32.0)	0.065	0.799	14.5(3.1-25.8)	1.254	0.267	14.4(3.3-25.5)	1.919	0.171
	BCS	22.2(10.1-34.2)			24.3(13.1-35.4)			26.3(15.4-37.3)		
Appetite loss (no)	Mastectomy	5.1(-5.9-16.3)	0.000	0.995	1.4(-5.3-8.3)	1.059	0.310	1.8(-3.1-6.8)	0.032	0.860
	BCS	5.2(-6.3-16.7)			7.5(0.4-14.6)			2.6(-2.6-7.7)		
Appetite loss (yes)	Mastectomy	10.2(3.3-17.0)	0.570	0.453	10.9(2.4-19.4)	0.164	0.687	5.6(-1.9-13.1)	0.484	0.489
	BCS	6.2(-0.4-12.9)			13.6(5.2-21.9)			9.6(2.2-17.0)		
Diarrhoea(no)	Mastectomy	0.4(-8.5-9.5)	0.420	0.521	2.6(-1.3-6.7)	0.498	0.484	1.3(-4.1-6.8)	0.475	0.494
	BCS	5.5(-6.4-14.8)			0.2(-3.9-4.4)			4.6(-1.0-10.3)		
Diarrhoea(yes)	Mastectomy	4.1(0.5-7.6)	1.252	0.268	4.7(-1.0-10.5)	0.247	0.621	1.2(-1.1-3.6)	0.041	0.839
	BCS	1.0(-2.4-4.5)			2.4(-3.2-8.2)			0.8(-1.5-3.1)		
Constipation (no)	Mastectomy	10.2(-4.7-25.2)	0.008	0.927	8.5(-0.5-17.5)	0.085	0.772	2.8(-3.3-8.9)	0.119	0.732
	BCS	9.0(-6.4-24.5)			6.2(-3.0-15.5)			4.6(-1.7-11.0)		
Constipation (yes)	Mastectomy	1.5(-5.5-8.6)	4.126	0.047	7.1(0.8-13.3)	0.435	0.512	4.4(-1.6-10.4)	0.890	0.349
	BCS	12.6(5.7-19.5)			10.2(4.1-16.3)			8.8(2.9-14.7)		
Financial difficulties (no)	Mastectomy	-1.1(-13.0-10.7)	2.933	0.095	4.4(-4.3-13.2)	0.153	0.697	2.3(-9.1-13.7)	1.101	0.300
	BCS	16.3(4.1-28.6)			7.6.4(-1-16.5)			12.7(0.8-24.5)		
Financial difficulties (yes)	Mastectomy	3.7(-3.6-11.1)	1.848	0.179	13.6(3.3-23.9)	0.091	0.764	14.5(2.9-26.1)	0.034	0.855
	BCS	11.5(4.2-18.8)			16.0(5.9-26.1)			16.1(4.7-27.6)		
**QLQ-BR23 Questionnaire**										
**Functional scales**^**a**^										
Body image (no)	Mastectomy	91.1(80.9-102.7)	0.006	0.938	97.7(90.3-105.1)	0.775	0.384	97.3(92.5-102.1)	0.021	0.886
	BCS	91.1(79.8-102.3)			92.1(84.4-99.7)			96.7(91.7-101.6)		
Body image (yes)	Mastectomy	93.1(88.0-98.3)	0.888	0.350	90.3(83.5-97.2)	0.009	0.927	89.8(82.7-96.9)	1.349	0.250
	BCS	97.0(91.8-102.3)			90.8(84.1-97.6)			96.1(89.2-103.1)		
Sexual functioning (no)	Mastectomy	18.7(6.3-31.1)	0.3	0.543	17.7(6.7-28.80	0.9	0.340	25.8(14.5-37.1)	5.9	0.020*
	BCS	12.2(-0.5-25.0)			8.6(-2.7-20.1)			2.4(-9.1-14.1)		
Sexual functioning (yes)	Mastectomy	18.1(9.3-26.7)	0.1	0.680	9.7(3.2-16.2)	0.1	0.758	8.9(2.8-15.1)	0.0	0.994
	BCS	15.3(6.7-23.8)			11.2(4.9-17.6)			8.9(2.9-15.0)		
Future perspective (no)	Mastectomy	69.3(52.4-86.2)	0.1	0.785	68.6(55.4-81.8)	2.7	0.105	67.7(52.6-82.7)	1.0	0.315
	BCS	65.3(47.9-82.8)			87.3(73.6-100.9)			80.7(65.2-96.2)		
Future perspective (yes)	Mastectomy	72.9(61.8-84.0)	0.1	0.744	70.1(58.8-82.5)	0	0.991	70.3(60.7-79.9)	2.2	0.139
	BCS	75.7(64.8-86.5)			70.8(59.1-82.4)			81.2(71.8-90.7)		
**Symptom scales**^**b**^										
Systemic therapy side effects (no)	Mastectomy	8.1(-1.5-17.6)	0.1	0.691	13.4(7.6-19.2)	1.1	0.2830	13.7(7.4-20.1)	0.456	0.504
	BCS	11.4(1.1-21.8)			7.9(1.7-14.1)			10.1(3.3-16.8)		
Systemic therapy side effects (yes)	Mastectomy	9.4(5.5-13.4)	0.2	0.619	11.4(4.9-17.9)	6.5	0.013*	9.0(4.8-13.1)	1.1	0.294
	BCS	7.9(4.1-11.8)			24.1(17.7-30.4)			12.3(8.2-16.3)		
Breast symptoms (no)	Mastectomy	14.9(2.1-27.6)	0.02	0.875	20.5(9.1-31.9)	2.2	0.140	10.3(3.5-17.1)	0.2	0.646
	BCS	13.1(0.1-26.3)			5.8(-5.9-17.6)			7.7(0.6-14.7)		
Breast symptoms (yes)	Mastectomy	10.9(4.3-17.5)	0.3	0.584	8.7(3.3-14.1)	0.7	0.405	13.1(7.6-18.5)	0.1	0.667
	BCS	8.2(1.7-14.6)			12.2(6.9-17.4)			11.3(5.9-16.6)		
Arm symptoms (no)	Mastectomy	14.9(2.1-27.6)	0.02	0.875	16.5(7.5-25.6)	0.9	0.328	7.4(-2.1-16.9)	3.4	0.070
	BCS	13.1(0.1-26.3)			8.9(-0.4-18.2)			22.5(12.6-32.4)		
Arm symptoms (yes)	Mastectomy	10.9(4.3-17.5)	0.3	0.584	9.5(2.5-16.5)	1.1	0.280	12.6(5.1-20.2)	0.1	0.767
	BCS	8.2(1.7-14.6)			8.4(8.4-22.2)			10.9(3.5-18.3)		

*P* values were calculated by Analysis of covariance (ANCOVA)

*Abbreviation*: *BCS =* Breast conserving surgery

^*^significant at *P* < 0.05

^a^Higher score indicates better functioning

^b^Higher score indicates more symptom

^c^ANCOVA adjusted for age, ethnicity hormonal therapy, radiotherapy and the quality of life score at time of diagnosis (baseline)

## Discussion

The current study found that Chinese (63.6%) and Indian (57.9%) women are more likely to have a mastectomy whereas Malay women received BCS (70%). At baseline, upon adjustment of demographical and clinicopathological factors, no significant difference were for QoL in both treatment groups. At 6 months, patients who underwent BCS had better social functioning scales (mean score 97.6 vs. 88.6 and *P* = 0.006). However, the differences in scores became insignificant at 1 year. BCS patients complained of worse symptoms, scoring a high scale for dyspnoea (mean score 12.5 vs. 2.5, and *P* = 0.031) at 6 months, compared to mastectomy patients. Even after stratification of chemotherapy, BCS patients who received chemotherapy seemed to experience more dyspnoea than mastectomy patients at 6 month follow-up period only. Although differences were present in social functioning and dyspnoea (symptom) scales at 6 months for both BCS and mastectomy groups, at 1 year, no statistical differences were noted for QoL between both groups. At one year mastectomy patients had better role functioning scores (mean score 94.8 vs.85.7, *P* = 0.029) compared to the BCS group. The effect remained even after stratification by chemotherapy treatment, we found mastectomy patients who only received chemotherapy had better role functioning compared to BCS group (mean score 95.2 vs.84.4 *P* = 0.034).

Baseline assessment revealed that sexual functioning was notably higher in the mastectomy group compared to the BCS group, with mean scores of 22.2 and 10.9, respectively (F = 5.2, *P* = 0.027). This disparity persisted at the six-month, with mean scores of 18.5 and 8.7 for mastectomy and BCS groups, respectively (F = 25.1, *P* = 0.000). However, after 1 year post-diagnosis, no significant difference in sexual functioning was observed. Upon further analysis, when stratified by chemotherapy data, mastectomy patients who did not undergo chemotherapy exhibited significantly higher mean scores for sexual functioning compared to the BCS group, but this difference was only apparent at the 1 year (mean score 25.8 vs. 2.4; F = 5.9, *P* = 0.02). After stratifying the chemotherapy data, it was observed that six months after diagnosis, BCS group patients experienced a higher occurrence of systemic therapy side effects in comparison to mastectomy patients, with mean scores of 24.1 and 11.4, respectively (F = 6.5, *P* = 0.013). However, no significant difference was noted in the 1 year.

The evaluation of the patient’s QoL, since the surgery is becoming progressively important in assisting decision-making concerning the types of management to be carried out, BCS or mastectomy [18]. The QoL of the study participants had high means functional scores (≥ 75 points) except for the sexual functioning scale (< 10 points) while low scores were recorded for symptom scores (< 26 points) in all time points. A similar trend was previously reported by a study done in Spain [19]. Breast cancer survivors can adjust well to their treatments and have low symptom scores, as the side effects are reversible [20, 21]. Notably at six months, there were insignificant differences in the functional scales between both groups, except for the social functioning scale (*P* < 0.001). Mastectomy patients need a longer time to adjust to their new body habitus, they may feel low self-confidence to return to normal living as seen in other Asian and Western studies [10, 16, 20, 21].

In contrast, BCS patients had significantly higher symptom scores for dyspnoea than mastectomized patients. After further classification, patients undergone chemotherapy seemed to experience more dyspnoea (*P* < 0.012). The presence of the breast may invoke more anxiety that may manifest as dyspnoea in panic attacks or anxiety attacks [22] There is evidence that women treated for breast cancer can have ongoing morbidity with symptoms of dyspnea, and reduced physical activity that can result in perceived poor health status [23].

Although it is a common belief that less-extensive surgeries would result in better cosmetic effects leading to maintenance of QoL and function [20]. On the other hand, at one year, there was no significant difference in the symptom scale between both groups. Previous literature had reported inconclusive findings related to QoL of breast cancer patients. Similarly, in Germany, a study assessing the QoL during the first 2 years following diagnosis using SF-12, found no difference in the QoL of patients treated with BCS and mastectomy [5]. Another study in Belgium reported significant benefits of BCS in compared to radical mastectomy surgery and mastectomy with reconstruction based on treatment satisfaction [9]. A study conducted in the USA found that patients undergoing BCS had better scores for satisfaction of their appearance and physical health (QoL domain) six months after surgery [4]. A longer follow-up (6–24 months) resulted in all three groups having identical QoL [4].

Furthermore, a study in Brazil examined clinical and demographic predictors affecting the QoL of breast cancer patients and found that worse QoL scores on physical and psychological scales were related to mastectomy [24]. In contrast, mastectomized patients undergoing chemotherapy tended to have better role-functioning scores (*P* < 0.034). Differences in QoL by geographical location may suggest different cultural nuances that we could not capture in our study. However, a possibility of diminishing cancer worry in mastectomy patients compared to BCS patients could explain better role functioning and lesser dyspnoea; as seen in a study from Australia that reported worse physical and role functioning scores among patients treated with conserving surgery [25].

However, in the current study, there was no significant association observed in physical functioning between both groups. Both groups routinely received physiotherapy and rehabilitation postoperatively in UMMC, thus optimal physical functioning was able to be achieved as the scores were quite high and physical functioning is not associated with the type of surgery.

A cross-sectional study done to assess QoL, revealed that women who had breast-conserving surgery reported a higher quality of life, improved sexual functioning, and fewer side effects from systemic therapy when compared to their counterparts who had mastectomy [26]. Factors such as the type of surgery, the age of the patient, and the time elapsed since the completion of treatment were identified as significant influencers of sexual functioning and quality of life in breast cancer survivors. Another study examining the impact of breast cancer treatments on short- and long-term sexual functioning, sexual enjoyment, and body image, and comparing them with age-matched women in the Norwegian general population [27], found that the sexual functioning score was notably low among the cancer patients, which aligns with our findings. However, women who had undergone mastectomy exhibited a modest yet significantly lower level of sexual functioning compared to those who had breast-conserving surgery in the long term, and breast cancer survivors who underwent chemotherapy showed decreased sexual functioning in the first year following treatment, which differs from our findings. This discrepancy may be attributed to our BCS group having a lower mean baseline sexual functional score compared to the mastectomy group even before the surgical procedures were performed.

A systematic review also compared the QoL between patients who had undergone BCS and mastectomy. It found that only body image scored significantly better for the BCS patients, while other domains were inconclusive [21]. A study in Germany reported no difference in QoL domains between both groups (mastectomy versus BCS), but there was a lesser satisfaction for body image in patients who underwent mastectomy [28]. Although our BCS patients seemed to have better body image for this study at 1 year, the data was statistically insignificant. Another narrative review involved the QoL of patients who underwent radical mastectomy, breast-conserving therapy, or oncoplastic breast surgery and found that oncoplastic breast surgery is associated with better QoL compared to the other two groups [20]. However, this type of surgery was not included in our study.

Scores for QoL are directly associated to the consequences of treatment among cancer patients, particularly for breast cancer [18]. Moreover, better levels of QoL (physical and mental domains) tend to be associated with a more encouraging prognosis and survival of patients [25]. However, QoL tools are not routinely used to evaluate the effects of cancer surgery. In Malaysia, only a few cross-sectional studies have examined QoL among breast cancer patients [10, 16, 29], but none of them has conducted any longitudinal study or evaluated the QoL of patients who underwent different type of surgery. Ganesh et al. found a mean general health status of 65.7 (SD = 21.4) which is lower than that demonstrated in our study. This might be due to the inclusion of patients with late stages of cancer (stage 3 and 4) in their study [16].

The age of the patients at the time of diagnosis also effects the QoL of breast cancer patients [30]. Prior studies have revealed variances in the impact of breast cancer on QoL for different age brackets, compared to the current study [31, 32]. The variations may be the result of different follow-up durations. In addition, the categorization of the patients based on different age groups before the assessment of QoL would possibly result in variances. However, most studies have also used the EORTC QLQ-C30 and EORTC QLQ-BR23 as tools to measure QoL.

Several methodological strengths and limitations of this study warrant mention. This study is the first longitudinal study in Malaysia that aimed to assess the QoL of patients who underwent mastectomy and BCS. In addition, the prospective design allowed the comparison of QoL before and after surgery, which provided better evidence and understanding of different options for surgery among breast cancer patients. However, the relatively smaller sample size due to the inclusion of only stages 0, 1, and 2 is seen as a limitation. Despite the prospective manner of this study for one year, a longer study period would be more valuable. Another limitation is that due to significant advancements in breast cancer diagnosis and treatment, the EORTC QLQ-BR 30 questionnaire has been superseded by the EORTC QLQ-BR45 tool [33]. However, since our baseline recruitment began in 2012, it was not feasible to transition to the new questionnaire midway through the study. Lastly, to observe the difference between different types of surgery, other aspects that may affect results such as radiotherapy or socioeconomic conditions and mental health and satisfaction issues were not studied.

## Conclusions

Patients who underwent BCS had better social functioning but worse dyspnoea symptoms and sexual functional scores compared to the patients undergone mastectomy at six months. However, there was no significant difference in QoL except for better role functioning among the mastectomy group was seen at one year following diagnosis. After further stratification

BCS group of patients who received chemotherapy experienced increased dyspnea and systemic therapy side effects at 6 months after their diagnosis compared to those who underwent mastectomy. However, 1 year after diagnosis, mastectomy patients who received chemotherapy exhibited improved role functioning, while those who did not undergo chemotherapy treatment appeared to have better sexual functioning compared to patients who underwent chemotherapy. To ensure optimal quality of life for breast cancer survivors need to offer postoperative social and physical support and monitoring for cancer-related concerns and other symptoms.

## Data Availability

The datasets generated during and/or analyzed during the current study are available from the corresponding author on reasonable request.

## Abbreviations

BCS: Breast conservative surgery
QoL: Quality of life
EORTC QLQ-C30: European Organization for Research and Treatment of Cancer Core Quality of Life
